# Maternal infection in the context of actively managed prelabor rupture of membranes at term: An observational study

**DOI:** 10.1002/ijgo.71018

**Published:** 2026-04-18

**Authors:** Inês Martins, Inês Gomes Brou, Miguel Macedo, Luísa Pinto

**Affiliations:** ^1^ Faculdade de Medicina Universidade de Lisboa (Medical School, University of Lisbon) Lisbon Portugal; ^2^ Department of Obstetrics Gynecology and Reproductive Medicine, Santa Maria University Hospital Lisbon Portugal

**Keywords:** chorioamnionitis, endometritis, infection, prelabor rupture of membranes, term

## Abstract

**Objective:**

To assess maternal infection following prelabor rupture of membranes (PROM) at term in a setting where active management is recommended, and to identify associated risk factors.

**Methods:**

A retrospective cohort study was conducted including pregnant women admitted to a Portuguese tertiary care center where immediate induction of labor is recommended following term PROM. Women with singleton term pregnancies complicated by PROM, who delivered between January 2020 and July 2023, were included. The primary outcome was intrauterine maternal infection, defined as clinical chorioamnionitis and/or endometritis. A comparative analysis was conducted between groups, based on latency to delivery (≤12 h vs. >12 h). A multivariable logistic regression model was used to evaluate the influence of confounding factors on the association between latency and the primary outcome.

**Results:**

A total of 759 patients with singleton term pregnancies complicated by PROM were included, corresponding to a prevalence of 9.7%. Chorioamnionitis and/or endometritis occurred in 40 patients (5.3%), with significantly different rates between the up to 12 h and the more than 12 h latency groups (0.9% vs. 7.3%, *P* < 0.001). Latency of more than 12 h was independently associated with maternal infection (odds ratio 9.0; 95% confidence interval 2.1–39.2). Group B streptococcus colonization status was not associated with increased maternal infection risk. Neonatal outcomes were similar between groups.

**Conclusion:**

This study demonstrates the increased risk of maternal infection when latency exceeds 12 h following term PROM, supporting the rationale for active management strategies and highlighting the need to evaluate additional prophylactic measures.

## 1 INTRODUCTION

Prelabor rupture of membranes (PROM) is defined as rupture of the fetal membranes in the absence of labor. This condition complicates about 8% of term pregnancies.^1^

PROM results from an imbalance between membrane reinforcement and degradation mechanisms. Activation of degradative enzymes, local inflammation, and mechanical forces all contribute to membrane weakening and eventual rupture.^2^ The diagnosis of PROM is usually based on clinical history and speculum examination revealing amniotic fluid leaking from the external cervical os. Laboratory tests including the nitrazine test, the ferning test, and rapid diagnostic assays detecting amniotic fluid proteins can be used to confirm PROM when clinical findings are inconclusive.^3^, ^4^ The natural course of term PROM typically involves spontaneous onset of labor within 94–107 h of membrane rupture for most women.^5^, ^6^

Infection is the major complication of term PROM.^1^, ^5^ The rate of neonatal infection can be up to seven times higher after PROM, particularly when chorioamnionitis occurs.^7^ Chorioamnionitis is an infection of the membranes, amniotic fluid, placenta, and/or decidua, most often caused by the ascending spread of microorganisms from the vaginal or enteric flora.^8^, ^9^, ^10^ Reported incidence rates of chorioamnionitis vary substantially across studies due to differences in study design, diagnostic criteria (clinical versus histologic definitions), heterogeneity in the prevalence of risk factors, and evolving obstetric practices.^9^, ^10^ A meta‐analysis reported a pooled incidence of 4.1%, although the true global burden is likely underestimated.^11^

In term pregnancies, clinical chorioamnionitis was identified in approximately 6%–10% of PROM cases^12^; however, many cases are subclinical, with microbial invasion of the amniotic fluid detected in up to 30% of women with term PROM.^13^ Prolonged membrane rupture is a well‐established risk factor.^14^ The most commonly involved microorganisms are *Ureaplasma* species, but polymicrobial infections are present in 70% of cases.^14^ Chorioamnionitis is associated with increased risks of other maternal infectious complications, including endometritis, pelvic abscess and, rarely, septic shock.^10^, ^15^ It is also linked to dysfunctional labor, cesarean delivery, and uterine atony.^16^, ^17^ Chorioamnionitis accounts for approximately one‐third of all stillbirths.^18^ The most important fetal complications include the fetal inflammatory response syndrome, early‐onset neonatal sepsis, and perinatal death.^19^

In women with term PROM, two management approaches are commonly used: active management with induction of labor (IOL), or expectant management, which involves waiting for the spontaneous onset of labor.^20^, ^21^ The purpose of IOL is to reduce the risk of infection, which increases as the interval between PROM and birth lengthens.^5^, ^12^ Routine antibiotic prophylaxis has not been shown to confer overall benefits in either maternal or neonatal outcomes.^1^, ^22^ However, when latency exceeds 12 h, antibiotic administration may help to prevent chorioamnionitis and endometritis.^1^

Uncertainty remains, particularly regarding the impact of risk factors on maternal infection in settings where active management is routinely practiced. Therefore, we aimed to assess the risk of maternal infection after term PROM in a context where immediate IOL is advised, and to identify associated risk factors. This may help to clarify whether prolonged latency continues to pose a significant risk under active management protocols, and whether further refinements to current practice are warranted, eventually including prophylactic antibiotic therapy.

## 2 MATERIALS AND METHODS

### 2.1 Study design and setting

A comparative retrospective observational study was conducted at a Portuguese tertiary care center.

### 2.2 Population

The study included pregnant women with singleton gestations, admitted with a diagnosis of term (37^+0^ to 41^+6^ weeks) PROM, who gave birth between January 2020 and July 2023. The diagnosis of PROM was confirmed by clinical examination demonstrating amniotic fluid leakage. The time of membrane rupture was determined based on patient's report.

The selected timeframe was based on the availability of computerized data on the Obscare® platform. Non‐cephalic fetal presentations were not excluded because these pregnancies were also exposed to PROM and infectious risk.

### 2.3 Intervention

Clinical management of term PROM followed the previously published local protocol.^8^ Immediate IOL is recommended upon admission, unless a cesarean is clinically indicated. Cervical ripening was performed using either a vaginal dinoprostone device (10 mg, up to 24 h) or vaginal misoprostol capsules (25 μg every 4 h, up to five times). Adjusted doses of oxytocin were administered for labor induction when the Bishop score was 6 or more. Maternal tympanic temperature was monitored every 8 h. A complete blood count and C‐reactive protein test were performed 24 h after PROM and repeated every 12 h. Testing was anticipated if clinical signs of infection emerged. In the absence of events warranting immediate clinical evaluation, vaginal examination is performed at the start of IOL; at each vaginal administration of misoprostol; at the time of removal of the dinoprostone device; every 4 h during the first stage of labor (every 2 h during the active phase); and every hour during the second stage of labor.

Antibiotic regimen for chorioamnionitis was intravenous ampicillin (2 g every 6 h) and gentamicin (4.5 mg/kg every 24 h), continued until 48 h postpartum. In cases of cesarean delivery, intravenous clindamycin (900 mg every 8 h) was added. For cases of endometritis, treatment consisted of intravenous clindamycin (900 mg every 8 h) plus gentamicin (4.5 mg/kg once daily) for at least 48 h.

### 2.4 Comparison

A comparative analysis was performed between two groups defined by latency from PROM to birth: 12 h or less versus more than 12 h. Latency was defined as the duration, in hours, from the rupture of membranes to delivery.

### 2.5 Outcomes

The primary outcome was intrauterine maternal infection, defined as clinical chorioamnionitis and/or endometritis. The diagnosis of clinical chorioamnionitis according to the local protocol^8^ required maternal temperature (>38.5°C) plus at least two of the following factors: maternal tachycardia (>100 beats/min), fetal tachycardia (>160 beats/min), uterine tenderness, foul‐smelling amniotic fluid or vaginal discharge, or maternal leukocytosis (>15 000 cells/mm^3^) and elevated C‐reactive protein. Postpartum endometritis was diagnosed based on clinical findings, including required maternal temperature (>38.5°C), maternal tachycardia, uterine tenderness, foul‐smelling lochia, leukocytosis (>15 000 cells/mm^3^), and elevated C‐reactive protein.

The secondary outcome was severe early‐onset neonatal sepsis,^9^, ^10^ referring to an invasive bacterial infection with manifestations within the first 72 h of life and a positive blood or cerebrospinal fluid culture, requiring admission to the neonatal intensive care unit.

### 2.6 Data and statistical methods

Data were obtained from the Obscare® software platform. Variables included demographic characteristics (maternal age, body mass index [calculated as weight in kilograms divided by the square of height in meters], nationality, and education), obstetric characteristics (gestational age at PROM, latency time, Bishop score, mode of birth, parity, Group B streptococcus colonization, gestational or pregestational diabetes, HIV infection, autoimmune disease), maternal outcomes (diagnosis of chorioamnionitis, endometritis, number of vaginal examinations, intrapartum fever, and mortality); and neonatal outcomes (newborn weight, Apgar scores, early neonatal sepsis, respiratory distress syndrome, and mortality).

The prevalence of term PROM and the rates of chorioamnionitis and/or endometritis were calculated. Student *t*‐test and *χ* ^2^‐test were applied to compare continuous and categorical variables, respectively. Multiple logistic regression analysis was performed to evaluate the effect of confounders on the association between latency and maternal infection. Statistical analyses were conducted using IBM SPSS® Statistics 2024 software (IBM, Armonk, NY, USA). A *P*‐value less than 0.05 was considered statistically significant.

## 3 RESULTS

During the study period, 8510 births occurred in our institution, of which 8293 were singleton pregnancies and 7847 were term births. A total of 759 patients with singleton pregnancies were diagnosed with term PROM, representing an estimated prevalence of 9.7% among term singleton pregnancies; all were included in the analysis.

The mean latency time from PROM to birth was 21.3 ± 14.8 h, with 235 patients delivering within 12 h and 524 after 12 h.

Baseline characteristics are presented in Table 1. As expected, primiparity was more frequent in the longer than 12 h latency group. Breech presentation and previous cesarean delivery were more frequent in the up to 12 h latency group, as cesarean delivery was often the elected mode of delivery in these cases following PROM. No other significant baseline differences were observed. Information on the number of vaginal examinations and the Bishop score at admission was not consistently available in extractable form.

**TABLE 1:** Maternal and obstetric characteristics^a^.

Characteristic	Group ≤12 h (*n* = 235)	Group >12 h (*n* = 524)	*P*‐value
Maternal age, y	31.26 ± 5.951	30.67 ± 5.853	0.200
Higher education	90 (38.3%)	214 (40.8%)	0.509
No higher education	145 (61.7%)	310 (59.2%)	
BMI	25.0 ± 5.0	24.8 ± 4.5	0.574
Gestational age, week	39.0 ± 1.077	39.2 ± 1.1	0.103
Primiparas	93 (39.6%)	359 (68.5%)	<0.001
Multiparas	142 (60.4%)	165 (31.5%)	
Previous cesarean delivery	27 (11.5%)	46 (8.8%)	0.031
Gestational diabetes	17 (7.2%)	31 (5.9%)	0.490
Pregestational diabetes	1 (0.4%)	2 (0.4%)	0.929
Positive HIV	3 (1.3%)	6 (1.1%)	0.877
Autoimmune disease	2 (0.9%)	8 (1.5%)	0.450
Group B streptococcal colonization	28 (11.9%)	88 (16.8%)	0.084
Cephalic presentation	90.2% (212)	98.5% (516)	<0.001
Breech presentation	9.8% (23)	1.5% (8)	

Abbreviation: BMI, body mass index (calculated as weight in kilograms divided by the square of height in meters).

^a^ Data are expressed as mean ± standard deviation and number (percentage).

Most women in the up to 12 h group (*n* = 183; 77.9%) experienced spontaneous labor onset after PROM; consequently, IOL was more frequent in the over 12 h group (Table 2). It is important to note that IOL did not prolong latency; rather, it reflects the indication for IOL in cases without early spontaneous labor. Overall, among women with spontaneous labor onset (*n* = 412; 54.3%), 55.6% had a latency period greater than 12 h (17 ± 13 h). Among those who underwent IOL after admission (*n* = 318; 41.9%), 87.4% delivered after 12 h (27 ± 15 h). The time from PROM to initiation of IOL was 4.6 ± 1.9 versus 9.2 ± 6.1 h (*P* < 0.001) in the up to 12 h group versus the over 12 h group, respectively, largely as the result of differences in the time from PROM to admission (median 1.7 vs. 2.9 h). The overall median time from admission to initiation of IOL was 5.8 h (range 0.5–11 h). Delays were mainly the result of operational factors, such as bed availability in the labor ward and the number of patients undergoing IOL. In women who underwent cervical ripening, the use of dinoprostone (45% [*n* = 18] vs. 45.3% [*n* = 126], *P* = 0.969) and misoprostol (42.5% [*n* = 17] vs. 43.2% [*n* = 120], *P* = 0.937) was similar in the up to 12 h and greater than >12 h latency groups.

**TABLE 2:** Obstetric and neonatal outcomes according to latency period after PROM (≤12 h vs. >12 h)^a^.

Outcome	Group ≤12 h (*n* = 235)	Group >12 h (*n* = 524)	*P*‐value
Latency between PROM and birth, h	8.0 ± 2.901	27.2 ± 14.2	<0.001
Chorioamnionitis and/or endometritis	2 (0.9%)	38 (7.3%)	<0.001
Intrapartum fever	6 (2.6%)	51 (9.7%)	<0.001
Spontaneous labor	183 (77.9%)	229 (43.7%)	<0.001
Labor induction	40 (17.0%)	278 (53.1%)	
Without labor	12 (5.1%)	17 (3.2%)	
Vaginal birth	173 (73.6%)	407 (77.7%)	0.224
Cesarean delivery	62 (26.4%)	117 (22.3%)	
Newborn weight, g	3218.6 ± 416.0	3207.2 ± 444.3	0.740
Apgar at 1 min	8.9 ± 1.3	8.9 ± 1.2	0.824
Apgar at 5 min	9.8 ± 1.0	9.8 ± 0.7	0.604
Apgar at 10 min	9.9 ± 0.9	9.9 ± 0.5	0.304
NICU	6 (2.6%)	12 (2.3%)	0.826
Neonatal severe early‐onset sepsis	0	2 (0.3%)	0.345
Neonatal RDS	4 (0.5%)	3 (0.4%)	0.136
Neonatal death	1 (0.1%)	1 (0.1%)	0.562

Abbreviations: NICU, neonatal intensive care unit; PROM, prelabor rupture of membranes; RDS, respiratory distress syndrome.

^a^ Data are expressed as mean ± standard deviation and number (percentage).

Forty patients (5.3%) developed clinical chorioamnionitis and/or endometritis, with a significantly higher rate in the over 12 h latency group (7.3%) than in the up to 12 h group (0.9%, *P* < 0.001). No maternal deaths occurred.

A multiple logistic regression model was constructed to analyze the influence of potential confounders on the association between latency and infection. Variables included maternal age, body mass index, parity, previous cesarean, Group B streptococcus colonization, maternal immunosuppressive conditions (gestational or pregestational diabetes, HIV infection, autoimmune disease), IOL, and mode of delivery. After adjustment, differences between groups in parity and IOL did not significantly influence infection rates. However, latency greater than 12 h (odds ratio 9.0, 95% confidence interval [CI] 2.1–39.2) was independently associated with chorioamnionitis and/or endometritis.

Neonatal outcomes were similar between groups. One neonatal death occurred in each group. In the up to 12 h latency group, a uterine rupture occurred in a woman with a previous cesarean who underwent spontaneous labor after term PROM. An emergency cesarean was performed due to non‐reassuring fetal status, but the infant died on day 23 from severe hypoxic–ischemic encephalopathy and refractory seizures. In the greater than 12 h latency group, a primigravida with spontaneous labor after PROM developed chorioamnionitis and a non‐reassuring fetal status. An emergency cesarean was performed, but the newborn died on day 2 from multiorgan dysfunction. Placental examination confirmed chorioamnionitis, with abscessed areas identified in the membranes overlying the placenta.

## 4 DISCUSSION

Our findings reveal a 9.7% prevalence of PROM in singleton term pregnancies and a higher rate of clinical chorioamnionitis and/or endometritis among women with prolonged latency. Although active management is standard practice, a latency period longer than 12 h increased the risk of maternal infection by nearly nine times.

After correct reinterpretation of the landmark TermPROM trial,^6^ evidence suggests that, in the natural course of term PROM, spontaneous birth occurs in half of women within 33 h and in 95% within 94–107 h, with IOL reducing the risk of maternal infection.^5^ Although the rationale for IOL is to reduce infection primarily by shortening latency, it has also been proposed that it may contribute to increased risk of infection due to more frequent vaginal examinations and medical interventions.^12^, ^23^, ^24^ Our records accounting for this intervention were not extractable; however, a higher number of vaginal examinations would be expected in the longer latency group, given its nature and the greater proportion of women undergoing IOL. Likewise, women in the longer latency group (composed of more nulliparous women) may have had lower Bishop scores at admission; nevertheless, cervical ripening rates were similar between groups. As adjustments for parity and mode of labor onset were not significant, a major impact of these potential confounders seems unlikely. The rate of cesarean delivery was similar between groups; however, cesarean delivery emerged as an independent risk factor for infection after adjustment. This is consistent with its known association with postpartum endometritis,^12^ ^,^ ^25^ and with the fact that chorioamnionitis often leads to labor arrest requiring cesarean delivery.

A Cochrane meta‐analysis previously revealed that IOL within the first 24 h after term PROM decreased maternal infection rate (relative risk [RR] 0.49, 95% CI 0.33–0.72), although the overall quality of evidence was low.^26^ A more recent secondary analysis of the TermPROM study^27^ demonstrated that IOL within the first 15–20 h was associated with a lower risk of maternal infection compared with expectant management. What stands out in our findings is that, even with the implementation of immediate IOL, most women (69%) experienced a latency greater than 12 h, and intrauterine infection remained more frequent in this subgroup.

Compared with a 13% rate of chorioamnionitis in a previous randomized controlled trial from our center,^28^ this study identified a much lower overall maternal infection rate of 5.3%. In that earlier study, immediate IOL was not yet standard practice and infections occurred exclusively in women with latency periods longer than 12 h.

With the implementation of immediate IOL after term PROM, a latency period longer than 12 h remains associated with an increased risk of maternal infection, stressing the need to evaluate other prophylactic measures, and eventually antibiotic prophylaxis for this subgroup of women. The meta‐analysis by Saccone and Berghella,^1^ which included the study from our center,^28^ revealed that in women with latency longer than 12 h, antibiotic prophylaxis significantly reduced the incidence of chorioamnionitis (6.1% vs. 2.9%, RR 0.49, 95% CI 0.27–0.91) and endometritis (0% vs. 2.2%, RR 0.12, 95% CI 0.02–0.62). Antibiotic regimens and timing differed across studies, with ampicillin and gentamicin used in the study conducted at our center.

A later retrospective cohort study comparing women with term PROM who were initiated on prophylaxis with cephalosporin found no statistical difference in efficacy or cost‐effectiveness between administration within versus after 6–12 h following rupture of membranes.^29^ Considering the role of *Ureaplasma* species as a cause of intra‐amniotic infection, future studies should consider the administration of antibiotics effective against these microorganisms, such as azithromycin or clarithromycin.^14^

A limitation of this study is its retrospective nature, using a computerized database. Relevant data such as the number and timing of vaginal examinations, Bishop score at admission, or exact duration of IOL were therefore unavailable for analysis. On the other hand, inclusion of cases from real‐world clinical practice, in the setting of a tertiary care center with a clearly defined management protocol, contributes to a better understanding of infection complicating term PROM.

In conclusion, there is a higher risk of maternal infection after term PROM when latency exceeds 12 h, even when active management is instated. The need to study other prophylactic measures should be evaluated.

## AUTHOR CONTRIBUTIONS

IM designed the work and contributed to acquisition, analysis and interpretation of data; formal analysis; and writing—original draft, review, and editing. IGB contributed to acquisition and interpretation of data and to writing—original draft and editing. MM contributed to acquisition and interpretation of data and to writing—editing. LP contributed to formal analysis and to writing—review and editing; and validated and supervised the study.

## CONFLICT OF INTEREST STATEMENT

The authors have no conflicts of interest.

## ETHICS COMMITTEE APPROVAL

The study received approval from the local ethics committee (Centro Académico de Medicina de Lisboa [CE‐CAML], no. 323/25). Informed consent was not obtained because of the retrospective nature of the study and practical constraints; however, all data were anonymized.

## Data Availability

Research data are not shared.
